# Artificial RNA Motifs Expand the Programmable Assembly between RNA Modules of a Bimolecular Ribozyme Leading to Application to RNA Nanostructure Design

**DOI:** 10.3390/biology6040037

**Published:** 2017-10-30

**Authors:** Md. Motiar Rahman, Shigeyoshi Matsumura, Yoshiya Ikawa

**Affiliations:** 1 Department of Chemistry, Graduate School of Science and Engineering, University of Toyama, Gofuku 3190, Toyama 9308555, Japan; d1681204@ems.u-toyama.ac.jp (Md.M.R.); smatsumu@sci.u-toyama.ac.jp (S.M.); 2 Graduate School of Innovative Life Science, University of Toyama, Gofuku 3190, Toyama 9308555, Japan

**Keywords:** catalytic RNA, group I intron, ribozyme, RNA nanostructure, RNA nanotechnology

## Abstract

A bimolecular ribozyme consisting of a core ribozyme (ΔP5 RNA) and an activator module (P5abc RNA) has been used as a platform to design assembled RNA nanostructures. The tight and specific assembly between the P5abc and ΔP5 modules depends on two sets of intermodule interactions. The interface between P5abc and ΔP5 must be controlled when designing RNA nanostructures. To expand the repertoire of molecular recognition in the P5abc/ΔP5 interface, we modified the interface by replacing the parent tertiary interactions in the interface with artificial interactions. The engineered P5abc/ΔP5 interfaces were characterized biochemically to identify those suitable for nanostructure design. The new interfaces were used to construct 2D-square and 1D-array RNA nanostructures.

## 1. Introduction

Nanometer-sized biomolecules and their assemblies with precisely controlled shapes are attractive materials for the expanding field of nanobioscience, which includes nanomedicine and nanobiotechnology. Nucleic acids (DNA, RNA, and their derivatives) are among the most promising biomaterials for nanobioscience purposes [1,2,3,4]. Nucleic acid nanotechnology is a rapidly developing research field as a result of the introduction of the DNA origami technique, first reported in 2006 [5], with which a variety of 2D- and 3D-shaped structures and assemblies can be constructed according to the established algorithm for structure design [1,2,5,6]. As single-stranded DNAs are used as components for the DNA origami technique, this technique can be applied to RNA with minimal methodology modifications [7,8,9,10]. Polygonal and polyhedral RNA nanostructures, which may be used as molecular cargos to deliver siRNAs, have also been constructed using several design strategies [11,12,13,14,15].

RNA is synthesized enzymatically in single-stranded form in the cellular environment, and several classes of RNAs, such as ribozymes and riboswitches, exhibit their functions after folding into defined 3D structures. These properties suggest that artificial RNA nanostructures designed by RNA origami (a methodology analogous to DNA origami) could be constructed in vivo [16]. To expand the methodologies of RNA nanostructure construction beyond RNA origami, we have recently reported the engineering of naturally occurring RNA structures [17]. This methodology allows us to install the biological functions of naturally occurring ribozymes and riboswitches into assembled RNA nanostructures, in which the biological functions can be expressed depending on the assembly of unit-RNA molecules. As a model system for this methodology, we employed a bimolecular group I ribozyme that was derived from the unimolecular *Tetrahymena* group I ribozyme and served as a structural platform.

The bimolecular group I ribozyme consists of a core ribozyme module (ΔP5 RNA) and an activator RNA unit (P5abc RNA), which form a complex expressing the full catalytic activity of the ΔP5 core ribozyme [18]. P5abc RNA binds tightly to ΔP5 RNA and stabilizes the catalytically active conformation of the ΔP5 core ribozyme [18,19]. Through a rational strategy, we connected P5abc RNA and ΔP5 RNA to design a unit RNA that can exhibit self-oligomerization. In the unit RNA, the two modules were joined by a connector element to allow the resulting unit to form closed oligomers with polygonal shapes, such as triangular trimers and square tetramers [17]. Self-oligomerization of the unit RNA yielded a mixture of assembled structures containing both triangular trimers and square tetramers. The selective formation of the desired structure was achieved by designing orthogonal interfaces between P5abc and ΔP5 modules [17,20]. In the orthogonal interfaces, we employed a non-natural RNA–RNA interacting motif, i.e., GGAA/R(GGAA), which belongs to the same RNA motif family (GNRA/receptor motif family) as the natural counterpart GAAA/R(GAAA) [21,22].

To increase the structural variety of the assembled RNA nanostructures, the repertoire of RNA–RNA interacting motifs is important because it governs the scope of RNA nanostructure design and assembly. In this study, we exploited RNA–RNA interacting motifs that can be used for RNA nanostructure assembly. We examined artificial RNA–RNA interacting motifs structurally unrelated to the GNRA/receptor family but that act as modular parts to functionally substitute for GNRA/receptor motifs in unimolecular ribozymes.

## 2. Materials and Methods

### 2.1. Plasmid Construction and RNA Preparation

Plasmids encoding mutants of ΔP5 RNA and P5abc RNA were generated by PCR-based mutagenesis of pTZ-ΔP5 and pP5abc [23], respectively. Plasmids encoding Lα RNA, Lβ RNA, F RNA, and their mutants were also derived from ΔP5 RNA by PCR-based mutagenesis. These plasmids were then used as templates for PCR to produce DNA fragments for in vitro transcription. The T7 promoter sequence was attached by PCR to a T7 promoter-containing sense primer. Transcription reactions were performed for 4.5 h at 37 °C in the presence of nucleotide triphosphates (1 mM each) and Mg^2+^ (15 mM). The DNA template in the reaction mixture was removed by DNase treatment. The transcribed RNA was purified in a 6% or 9% denaturing polyacrylamide gel. A published protocol was used for 3′-end labeling of RNAs with the BODIPY-fluorophore synthesized in our laboratory [24].

### 2.2. Electrophoretic Mobility Shift Assay (EMSA)

Electrophoretic mobility shift assays (EMSA) for bimolecular P5abc/ΔP5 complexes were performed according to the procedure reported by Tanaka et al. [20]. EMSAs were carried out to analyze the assembly of Lα RNA, Lβ RNA, and F RNA according to the procedure reported by Oi et al. [17].

### 2.3. Ribozyme Activity Assay

Aqueous solutions containing ΔP5 RNA (final concentration: 0.5 μM) and P5abc RNA (final concentration: 0.75 μM) were heated separately at 80 °C for 5 min and then cooled to 37 °C. The 10× concentrated reaction buffer (final concentrations: 30 mM Tris-HCl, pH 7.5, and given concentration of MgCl_2_) containing 2 mM guanosine triphosphate (final concentration: 0.2 mM) was added to the RNA solutions, which were further incubated at 37 °C for 30 min. Ribozyme reactions were initiated by adding 5′-FAM-labeled substrates (final concentration: 0.5 μM) and allowed to proceed at 37 °C. Aliquots were taken at given time points and treated with 1.5 volumes of stop solution containing 75% formamide, 0.1% xylene cyanol, and 100 mM EDTA. The mixtures were electrophoresed in 15% denaturing polyacrylamide gels. The gels were then analyzed with a Pharos FX fluoroimager (BioRad, Hercules, CA, USA). All assays were repeated at least twice. The mean values are plotted in the figures, with the minimum and maximum values indicated by error bars.

## 3. Results and Discussion

### 3.1. Assembly of Bimolecular Group I Ribozymes through Artificial RNA–RNA Interacting Motifs

To determine the structural and functional modularity of artificial RNA–RNA interacting motifs that were generated through in vitro selection experiments, we investigated their abilities to act as modular parts for the assembly of the bimolecular P5abc/ΔP5 ribozyme derived from the *Tetrahymena* group I intron. For biochemical characterization of the bimolecular P5abc/ΔP5 ribozyme, simple methods have been established that allow to estimate the physical assembly between the two module RNAs, as well as the catalytic ability of the resulting complex [20,25]. It was shown that three RNA–RNA interacting motifs could functionally substitute for the naturally occurring GNRA/receptor interacting motifs in unimolecular ribozymes [26,27,28]. We examined the three artificial motifs as alternative motifs to substitute the GNRA/receptor interacting motif supporting the intermolecular assembly of P5abc RNA and ΔP5 RNA to conduct catalysis.

**Figure 1:**
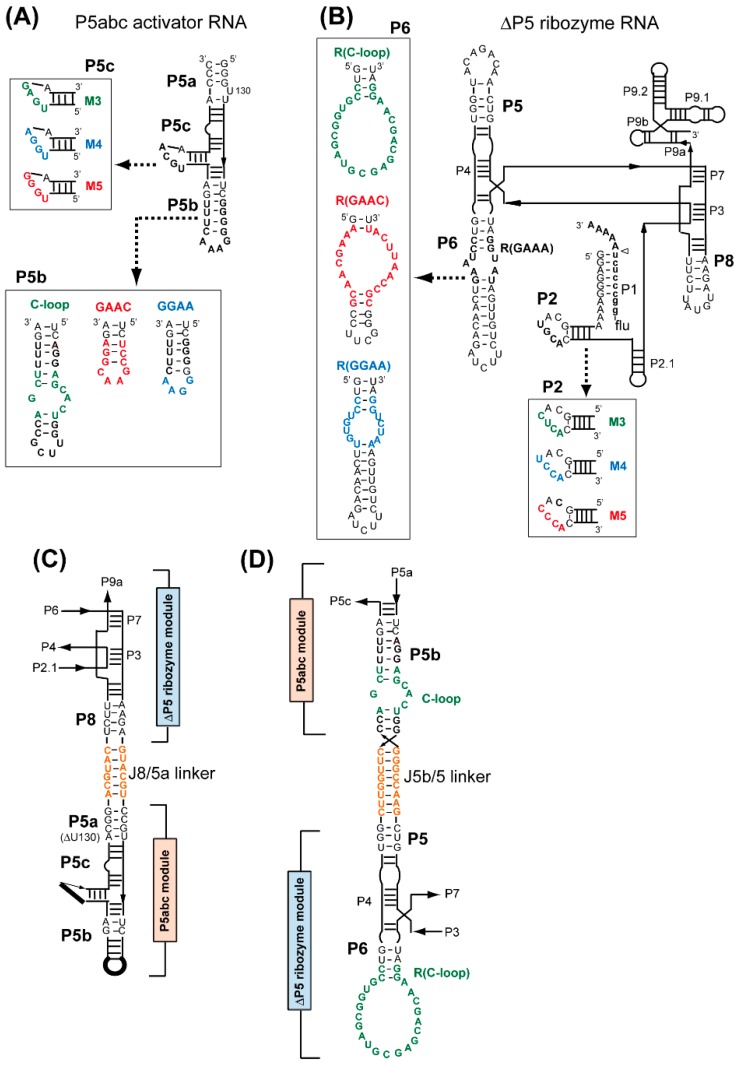
The activator RNA unit (P5abc RNA) and the core ribozyme module (ΔP5 RNA) were used to form a bimolecular group I ribozyme and engineered group I ribozymes for the assembly of RNA nanostructures. (**A**) Secondary structures of P5abc RNA and its variants used in this study. (**B**) ΔP5 RNA corresponding to the core structure of the *Tetrahymena* group I ribozyme. (**C**) Partial structure of Lα RNA and Lβ RNA showing the modular organization of the ΔP5 module and P5abc module, which are connected at P8 and P5a via a duplex linker. (**D**) Partial structure of F RNA showing the modular organization of the ΔP5 module that presents the R(C-loop) motif and the P5abc module with the C-loop motif. The two modules are connected at P5 and P5b via a duplex linker. In F RNA, the original 5′ and 3′ ends of the C-loop P5abc RNA (see Figure 1A) were capped with a stable 5′-UUCG-3′ tetraloop.

**Figure 2:**
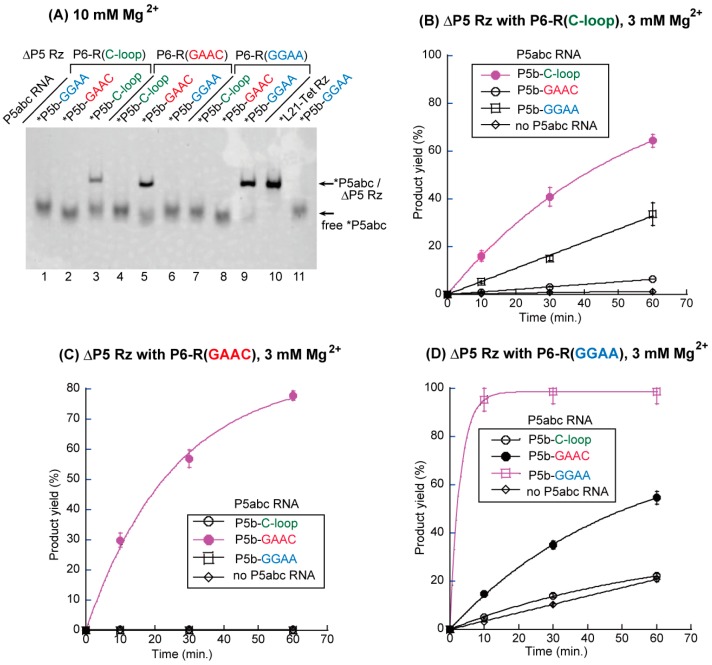
Biochemical analyses of RNA–RNA interacting motifs that alternatively support the P5b–P6 interaction in the P5abc/ΔP5 bimolecular ribozyme. (**A**) Electrophoretic mobility shift assay (EMSA) of the P5abc/ΔP5 complexes in the presence of 10 mM Mg^2+^. L21-Tet Rz RNA (the parental unimolecular ribozyme from which the P5abc/ΔP5 complex was derived) and P5b-GGAA RNA were used as size markers for the complex and free-P5abc RNA, respectively. The asterisks indicate RNAs labeled with the BODIPY fluorophore. (**B**–**D**) Time courses of substrate-cleavage reactions catalyzed by the P5abc/ΔP5 bimolecular ribozymes in the presence of 3 mM Mg^2+^. Some error bars are small enough to be hidden by the symbols.

We constructed three sets of P5abc/ΔP5 pairs: the variant P5abc RNAs possessed the C-loop motif, the GAAC loop motif, or the GGAA loop motif in P5b region, substituting for the parent GAAA loop (Figure 1A); the variant ΔP5 RNAs possessed the R(C-loop) motif, the R(GAAC) motif, or the R(GGAA) motif in the P6 region, substituting for the parent R(GAAA) motif (Figure 1B). Among the three sets of interacting motifs, only the GGAA/R(GGAA) pair belonged to the GNRA/receptor family [21,22]. The relative binding affinity between P5abc RNA and ΔP5 RNA was evaluated by electrophoretic mobility shift assay (EMSA) through the analysis of the Mg^2+^ concentration-dependence of their complex formation. Mg^2+^ is known to act as a general stabilizer of RNA–RNA interactions [29,30]. Weaker (or mismatched) RNA–RNA interactions require higher Mg^2+^ ion concentrations to establish a P5abc/ΔP5 complex that appears as a retarded band [20,23]. We examined nine pairs of P5abc RNA and ΔP5 RNA, among which three pairs were matched P5b/P6 pairs (Figure 2A, lanes 3, 5, and 9), and the remaining six were mismatched P5b/P6 pairs.

EMSA with 10 mM Mg^2+^ showed complex formation in three P5abc/ΔP5 pairs with matched P5b–P6 interactions (Figure 2A, lanes 3, 5, and 9). The remaining six pairs with mismatched P5b–P6 interactions showed no retarded band, indicating that the disruption of the P5b–P6 interaction made the P5abc/ΔP5 complex unstable in the presence 10 mM Mg^2+^. The importance of the P5b–P6 interaction in P5abc/ΔP5 complex formation was confirmed by the observation that no mismatched complex was stably formed even with 15 mM Mg^2+^, although a very weak band was detectable in the mismatched P5b-GAAC/P6-R(GGAA) pair (Supplementary Materials Figure S1A). We then investigated the relative order of physical affinity among the three artificial RNA–RNA interacting motifs by reducing the Mg^2+^ ion concentration to 5 mM (Figure 2B). In EMSA with 5 mM Mg^2+^, the P5b-GGAA/P6-R(GGAA) pair formed a bimolecular complex. However, neither the P5b-GAAC/P6-R(GAAC) pair nor the P5b-C-loop/P6-R(C-loop) pair showed a retarded band (Supplementary Materials Figure S2B), indicating that the physical affinities of these two matched pairs were not sufficiently high to stably form the P5abc/ΔP5 ribozyme complex in the presence of 5 mM Mg^2+^. These observations indicated that the three artificial interacting motifs are orthogonal to each other. On the other hand, in the structural context of the P5abc/ΔP5 complex, the binding affinities of the two non-GNRA/receptor interactions were lower than that of the GGAA/R(1) interaction belonging to the GNRA/receptor family.

To determine whether the P5abc/ΔP5 complexes supported by artificial motifs were catalytically active, their abilities to promote substrate-cleavage reactions were examined. A 1.5-fold excess molar amount of P5abc RNA (0.75 μM) was added to activate the ΔP5 core ribozyme in the presence of equimolar amounts of the substrate RNA (0.5 μM) and the ΔP5 RNA (0.5 μM). In the presence of 3 mM Mg^2+^, three biomolecular ribozymes possessing matched P5b–P6 interactions exhibited higher catalytic activity than the remaining six pairs possessing mismatched P5b–P6 interactions (Supplementary Materials Table S1). On the other hand, the activity of the ribozyme with the matched C-loop/R(C-loop) pair (kobs = 0.02 min^−1^, Figure 2B) was the same as that with a mismatched GAAC/R(GGAA) pair (kobs = 0.02 min^−1^, Figure 2D). The comparison of nine bimolecular ribozymes indicated that a ΔP5 RNA variant with an R(GAAC) motif showed the highest specificity to its cognate partner P5abc with a GAAC loop (kobs = 0.04 min^−1^), since the remaining two P5abc RNAs could not activate the core ribozyme (Figure 2C). A ΔP5 RNA variant with an R(GGAA) motif exhibited the highest catalytic activity in the presence of its cognate partner (kobs = 0.34 min^−1^), among the three matched complexes. On the other hand, the P6-R(GGAA) variant was also moderately activated by a mismatched P5abc with a GAAC loop (Figure 2D). This was consistent with the results of EMSA in the presence of 15 mM Mg^2+^, in which the mismatched GAAC/R(GGAA) pair weakly supported the P5abc/ΔP5 complex (Supplementary Materials Figure S1A). The C-loop/R(C-loop) pair was the least active pair among the three matched pairs in terms of both the binding specificity of P5abc and ΔP5 RNA and the catalytic activity of the matched P5abc/ΔP5 complex. 

### 3.2. Artificial Kissing Loop Interactions to Assemble Bimolecular Group I Ribozymes

It has been reported that complex formation of P5abc RNA and ΔP5 RNA is supported not only by the RNA–RNA interaction between P5b and P6, but also by four Watson–Crick base pairs between P5c in P5abc RNA and P2 in ΔP5 RNA [20,31]. The P5c–P2 interaction has been shown to govern P5abc/ΔP5 complex formation cooperatively with, and dominantly over the P5b–P6 interaction [23]. To engineer the binding specificity between P5abc RNA and ΔP5 RNA, we generated an artificial P5c/P2 base pair termed M3 (Figure 1A) [20]. The M3 pair was orthogonal to the parent P5c/P2 pairs, but the P5abc/ΔP5 complex supported by M3 was less stable than the parent P5abc/ΔP5 complex [20]. Employing the new P5c/P2 base pairs in combination with three P5b–P6 interactions, the repertoire of P5abc/ΔP5 interfaces can be expanded, and some of the interfaces would be orthogonal to each other. 

**Figure 3:**
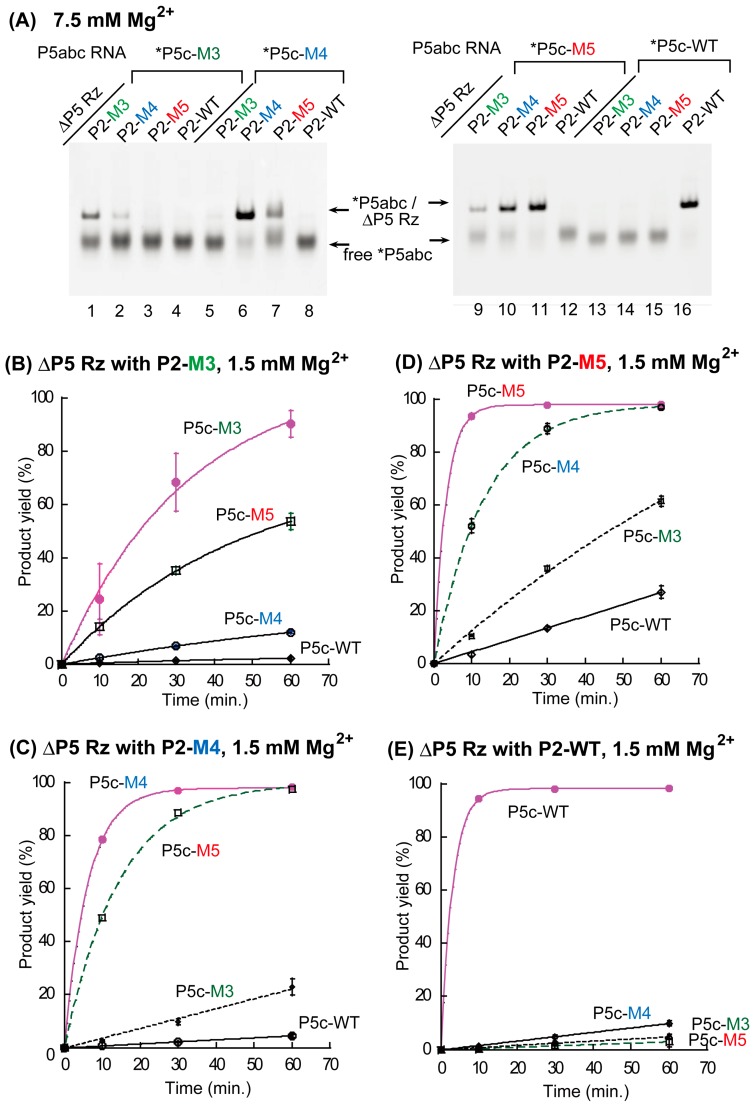
Biochemical analyses of the base pairs that can substitute the parental P5c–P2 base-pair interaction in the P5abc/ΔP5 bimolecular ribozyme. (**A**) EMSA of the P5abc/ΔP5 complexes in the presence of 7.5 mM Mg^2+^. The asterisks indicate RNAs labeled with the BODIPY fluorophore. (**B–E**) Time courses of substrate-cleavage reactions catalyzed by the P5abc/ΔP5 bimolecular ribozymes in the presence of 1.5 mM Mg^2+^. Some error bars are small enough to be hidden by the symbols.

In this study, we designed two new P5c/P2 base pairs (M4 pair and M5 pair, see Figure 1A) and compared their properties with the previously characterized M3 pair and with the parental, wild-type (WT) pair, by EMSA and a catalytic activity assay of the P5abc/ΔP5 complex (Figure 3). Although the design of M4 and M5 pairs was primarily focused on the orthogonality to the WT pair, we also examined all the 16 possible combinations of P5c/P2 base pairs (Supplementary Materials Figure S2) by EMSA and the activity assay. In the presence of 5 mM Mg^2+^ (Supplementary Materials Figure S3) and 7.5 mM Mg^2+^ (Figure 3A), two matched P5c/P2 base pairs (WT and M5) stably supported the P5abc/ΔP5 complex (Figure 3A and Supplementary Materials Figure S3, lanes 11 and 16). The M4 pair also formed the complex, but free P5abc RNA remained (Figure 3A and Supplementary Materials Figure S3, lane 6). On the other hand, the M3 pair poorly supported the complex in the presence of 5 mM Mg^2+^ (Supplementary Materials Figure S3, lane 1), confirming the previous observation that the M3 pair interacted less strongly than the parental pair. While the M5 pair supported the complex more efficiently than both the M4 pair and the M3 pair (Supplementary Materials Figure S3), P5abc RNA with P5c-M5 (P5abc-M5) recognized its cognate ΔP5 RNA less selectively, because P5abc-M5 also formed complexes with mismatched ΔP5 RNAs containing P2-M3 and P2-M4 (Figure 3A, lanes 9 and 10). This property was expected, since, as shown in Figure S2, P5c-M5 forms four base pairs containing single G-U with P2-M3 and P2-M4. The formation of similar base pairs was also predicted between P5c-M5 and P2-M4, and the pair formed the corresponding complex at a modest degree (Figure 3A, lane 7).

We then investigated whether the binding affinity and the specificity of base pairs, which were predicted in Supplementary Materials Figure S2 and examined by EMSA (Figure 3A), correlated with the catalytic activity of P5abc/ΔP5 bimolecular ribozymes. In the presence of 1.5 mM Mg^2+^, each of the four ΔP5 RNAs exhibited its highest activity with its cognate partner P5abc (Table S2). The parent WT bimolecular ribozyme (*k*_obs_ = 0.32 min^−1^) and its M5 variant (*k*_obs_ = 0.31 min^−1^) showed closely similar activity, and were 2-fold more active than the M4 variant (*k*_obs_ = 0.16 min^−1^). The M3 variant (*k*_obs_ = 0.03 min^−1^) was distinctly less active than the three complexes. The rate constant of the M3 variant (0.03 min^−1^) was even smaller than those of two mismatched complexes having the P5c-M5/P2-M4 mismatched pair (Figure 2C, *k*_obs_ = 0.069 min^−1^) and the P5c-M4/P2-M5 pair (Figure 2D, *k*_obs_ = 0.076 min^−1^). These observations are qualitatively consistent with those from structure prediction and EMSA.

### 3.3. Formation of RNA Squares through Selective Assembly of Engineered Group I Ribozymes

**Figure 4:**
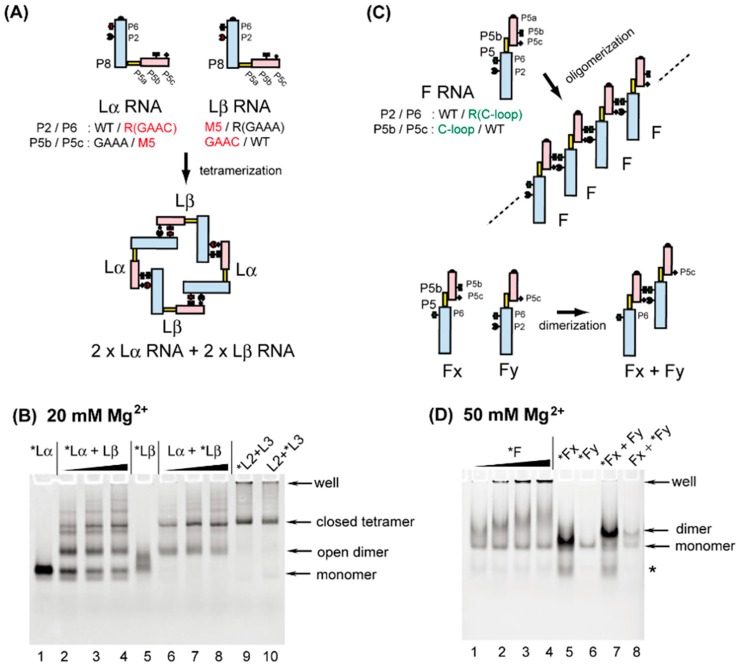
Oligomeric RNA nanostructures whose assembly was mediated by the association between the P5abc module and the ΔP5 module. (**A**) Selective formation of closed tetramers by Lα RNA and Lβ RNA. (**B**) EMSA of Lα RNA + Lβ RNA in the presence of 20 mM Mg^2+^. The asterisks indicate monomer RNAs labeled with the BODIPY fluorophore. In lanes 1 and 5, the concentrations of Lα and Lβ were 0.5 μM. The concentrations of Lα + Lβ were 0.5 + 0.5 μM in lanes 2 and 6, 1.0 + 1.0 μM in lanes 3 and 7, and 2.0 + 2.0 μM in lanes 4 and 8. The concentrations of L2 + L3 were 0.5 + 0.5 μM in lanes 9 and 10. (**C**) One-dimensional open oligomers of F RNA and selective dimerization of its mutants. (**D**) EMSA of F RNA and its mutants in the presence of 50 mM Mg^2+^. The asterisks indicate monomer RNAs labeled with the BODIPY fluorophore. The concentrations of F RNA were 0.25 μM, 0.5 μM, 1.0 μM, and 2.0 μM in lanes 1, 2, 3, and 4, respectively. The concentrations of Fx RNA and Fy RNA were 0.5 μM in lanes 5 and 6. The concentrations of Fx RNA + Fy RNA were 0.5 + 0.5 μM in lanes 7 and 8.

To design artificial RNA nanostructures with new P5b/P6 and P5c/P2 interacting pairs through modular engineering of the group I ribozyme, we chose the GAAC-R(GAAC) pair and the M5 pair as new modular parts for a closed RNA tetramer with square shape. Monomer units of this square tetramer are engineered variants of the group I ribozyme (Figure 4A) that we described previously [17]. In the monomer units, the P5a region of the P5abc module and the P8 region of the ΔP5 module were covalently bonded with a duplex serving as a rigid spacer (Figure 1C). The resulting unit RNAs, containing both P5abc and ΔP5 modules in a single RNA molecule, assembled intermolecularly to form oligomers of modular ribozymes [17]. Two engineered ribozymes were designed to alternately oligomerize, thus preferentially forming a closed tetramer. 

We have previously characterized the closed tetramer consisting of L2 RNA and L3 RNA. L2 RNA possesses P2-M3, P6-R(GAAA), P5c-WT, and P5b-GGAA, whereas L3 RNA possesses P2-WT, P6-R(GGAA), P5c-M3, and P5b-GAAA (see lanes 9 and 10 in Figure 4B). We replaced the M3 pair and the GGAA/R(GGAA) pair in L2 and L3 RNAs with the M5 pair and the GAAC/R(GAAC) pair, respectively. The resulting unit RNAs were designated as Lα RNA and Lβ RNA. Lα RNA possessed P2-WT, P6-R(GAAC), P5c-M5, and P5b-GAAA, while Lβ RNA possessed P2-M5, P6-R(GAAA), P5c-WT, and P5b-GAAC (Figure 4A).

In the presence of 20 mM Mg^2+^, L2 and L3 RNAs were shown to efficiently and selectively form a closed tetramer through the formation of an L2 + L3 heterodimer. The assembly behavior of L2 and L3 RNAs allowed us to employ them as a size marker for closed tetramers (Figure 4B, lanes 9 and 10). In the presence of 20 mM Mg^2+^, a major band was clearly seen in samples containing equimolar amounts of Lα RNA and Lβ RNA. The mobility of this major band was similar to that in L2 + L3 samples, suggesting that the band corresponds to a closed tetramer consisting of 2 × Lα RNA + 2 × Lβ RNA. The alternate oligomerization of Lα and Lβ was also supported by the mobility of Lα RNA and Lβ RNA in the absence of partner RNA. Lα RNA migrated as a single band (Figure 4B lane) corresponding to its monomeric form, whereas Lβ RNA showed a broad band (Figure 4B, lane 5), suggesting that Lβ RNA may have structural polymorphism in its monomeric state possibly because of misfolding. This possible structural polymorphism was dissolved upon assembly with Lα RNA to form an open heterodimer that was further converted to the closed tetramer (Figure 4B, lanes 6–8). The Lα + Lβ closed tetramer migrated slightly faster than the L2 + L3 closed tetramer, probably because the total number of nucleotides in the Lα + Lβ tetramer was 18 nucleotides smaller than that in the L2 + L3 tetramer.

The oligomerization of Lα RNA and Lβ RNA seemed to be less efficient than that of L2 and L3, because the conversion of the open dimer to the closed tetramer proceeded incompletely. No higher order oligomers, unable to enter the gel matrix, were observed in Lα RNA and Lβ RNA sample lanes. The design of Lα + Lβ assembly simply copied the established nanostructure of L2 + L3. Further investigation and modification of the L2 + L3 square and of the Lα + Lβ square would generate orthogonal sets of closed tetramers, whose combined use would expand the scope of ribozyme-based RNA nanostructures.

### 3.4. Formation of Novel RNA 1D Array through Selective Oligomerization

Biochemical characterization of the C-loop–R(C-loop) interaction in the context of the bimolecular ribozyme demonstrated that this interacting motif, which can be used as a possible alternative to the naturally occurring GNRA–receptor interactions [26], showed no advantage over the parent motif and two other artificial motifs, in terms of binding affinity or specificity between P5abc and ΔP5 modules (Figure 2). The secondary structures of the C-loop motif (internal loop, Figure 1A) and the R(C-loop) motif (terminal hairpin loop, Figure 1B) are the opposite of those of GNRA loops (terminal hairpin loops, Figure 1A) and their receptors (internal loops, Figure 1B) [26]. The unique structure of the C-loop/R(C-loop) interacting pair, however, enabled us to connect the P5abc module and ΔP5 RNA in a manner that would otherwise have been impossible (Figure 1D). We capped the original 5′ and 3′ ends of the C-loop P5abc RNA with a stable 5′-UUCG-3′ tetraloop and removed the UUCG loop in the C-loop motif (Figure 1A). This modified C-loop P5abc RNA was then connected to the P5 region of the ΔP5 RNA with the R(C-loop) in P6. The resulting unit RNA (designated as F RNA) was expected to self-oligomerize to form 1D open oligomers (Figure 4C). Similar design of 1D open oligomers has been reported by using tecto-RNA as a unit RNA [32].

In the presence of 50 mM Mg^2+^, the mobility of F-RNA was retarded gradually in an RNA concentration-dependent manner (Figure 4D, lanes 1–4). This type of mobility changes is usually caused by a dynamic equilibrium between monomeric and oligomeric states [33]. In addition, possible higher-order oligomers of F RNA, which could not enter the gel matrix, were also observed in an RNA concentration-dependent manner (Figure 4D, lanes 1–4). To test whether the retarded bands in lanes 1–4 resulted from self-oligomerization of F RNA, we prepared two mutant F RNAs (Fx and Fy), which had a UUCG loop in P2 and the M5 loop (Figure 1A) in P5c, respectively. Each RNA existed in the monomeric state because no retarded bands were formed in the absence of their partner RNAs (Figure 4D, lanes 5 and 6). In each sample lane containing both Fx RNA and Fy RNA, a single retarded band was observed (Figure 4D, lanes 7 and 8), which would correspond to the Fx + Fy heterodimer. Consistent with the molecular design, on the basis of which Fx and Fy RNAs can form only heterodimers, no oligomers larger than dimers were observed in lanes 7 and 8. The resulting 1D open oligomers of F RNA may be insufficiently stable because of the limited recognition ability of the C-loop–R(C-loop) interaction (Figure 2). The molecular design of F RNA may be potentially attractive to expand the nanostructure design based on large modular ribozymes.

## 4. Conclusions

We have characterized artificial RNA–RNA interactions to determine their abilities to support the assembly of the two module RNAs in the P5abc/∆P5 bimolecular ribozyme. Some of the new RNA–RNA interactions characterized in this study were used as alternative modular parts to produce new P5abc/∆P5 interfaces. These new interfaces can be applied to design ribozyme-based RNA nanostructures.

## Supplementary Materials

The following are available online at www.mdpi.com/2079-7737/6/4/37/s1, Figure S1: Electrophoretic mobility shift assay of P5abc/∆P5 complexes in the presence of 15 mM Mg^2+^ and 5 mM Mg^2+^, Figure S2: Possible base pairs between matched and mismatched combinations of P5c and P2, Figure S3: Electrophoretic mobility shift assay of P5abc/ΔP5 complexes in the presence of 5 mM Mg^2+^, Table S1: Effects of P5b-P6 interaction on observed rate constants of the bimolecular ribozymes, Table S2: Effects of P5c-P2 interactions on observed rate constants of the bimolecular ribozymes.
