# Supplementary material for: Artificial RNA Motifs Expand the Programmable Assembly between RNA Modules of a Bimolecular Ribozyme Leading to Application to RNA Nanostructure Design
Source: Biology (Basel). 2017 Oct 30;6(4):37. doi: 10.3390/biology6040037 (PMC5745442; doi:10.3390/biology6040037)
Supplement: Supplementary file 1 [file biology-06-00037-s001.pdf]

Supporting Information

Artificial RNA motifs expand programmable assembly between RNA modules of a bimolecular ribozyme leading to application to RNA nanostructure design

Md. Motiar Rahman, Shigeyoshi Matsumura and Yoshiya Ikawa

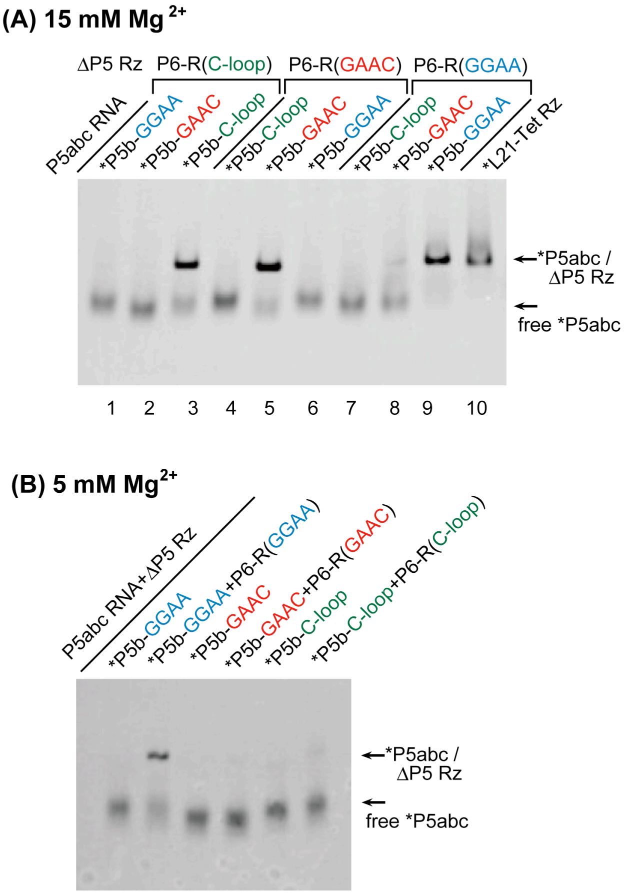

Figure S1

Electrophoretic mobility shift assay of P5abc/ΔP5 complexes in the presence of 15 mM Mg^{2+} (A) and 5 mM Mg^{2+} (B). L21-Tet Rz RNA (the parental unimolecular ribozyme from which the P5abc/ΔP5 complex was derived) and P5b-GGAA RNA were used as size markers for the complex and free-P5abc RNA, respectively. Asterisks indicate RNAs labeled with the BODIPY fluorophore.

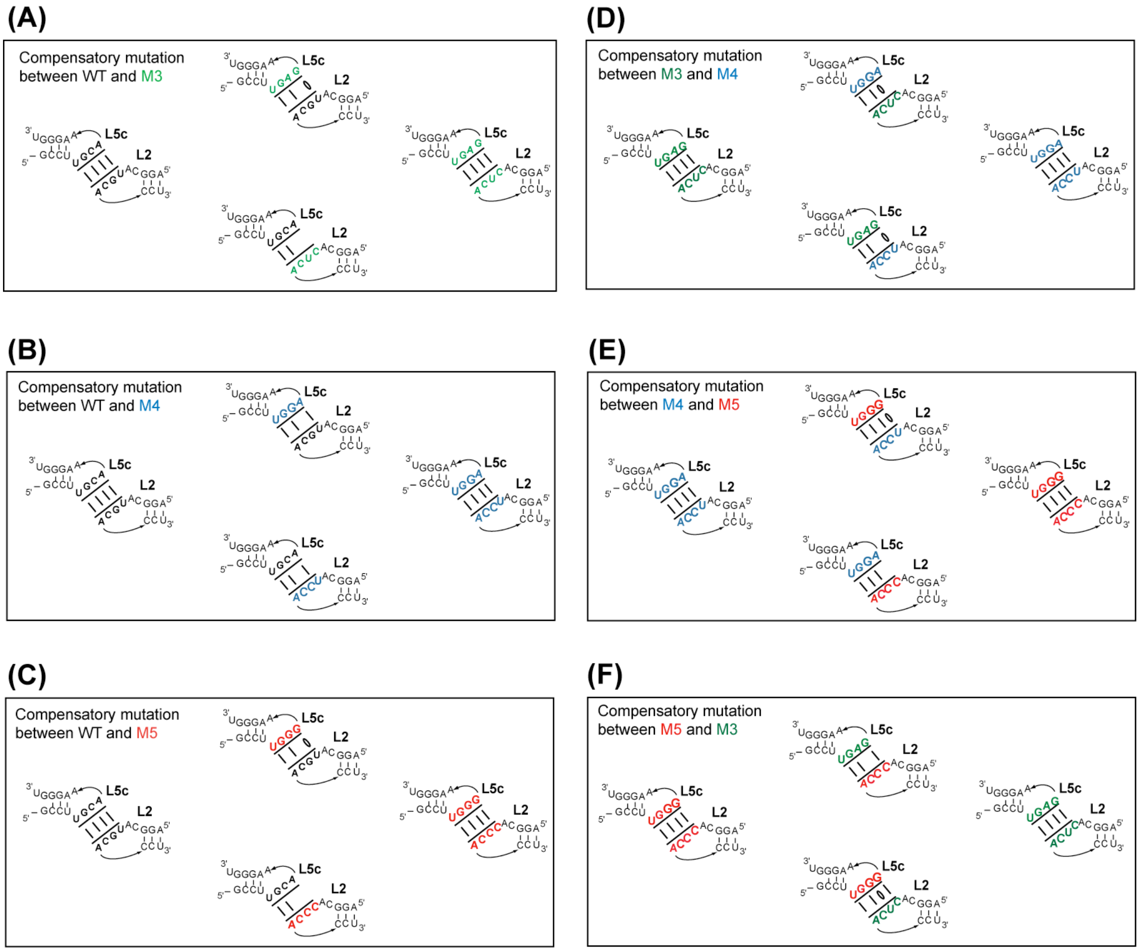

Figure S2

Possible base pairs between matched and mismatched combinations of P5c and P2. L5c and L2 indicate the loop regions of P5c and P2 elements, respectively.

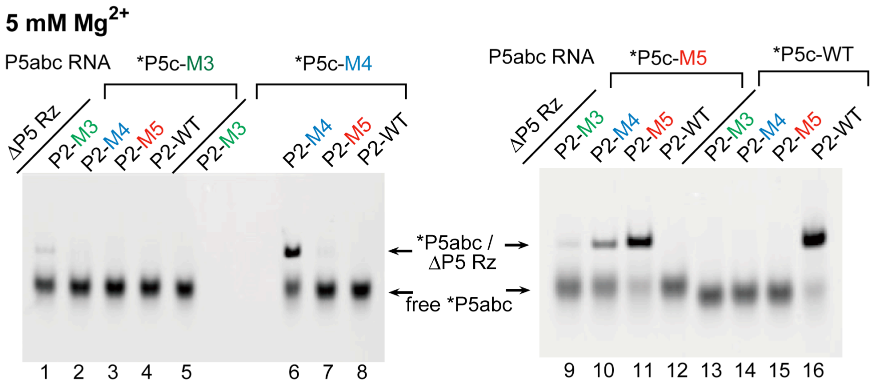

Figure S3
Electrophoretic mobility shift assay of P5abc/ΔP5 complexes in the presence of 5 mM Mg^{2+}. Asterisks indicate RNAs labeled with the BODIPY fluorophore.

Table S1
Effects of P5b-P6 interactions on observed rate constants of the bimolecular ribozymes.

P6 in ΔP5 ribozyme	P5b in P5abc RNA	k_{obs} (min^{-1})
R(C-loop)	C-loop	0.02
R(GAAC)	GAAC	0.04
R(GGAA)	GGAA	0.34
R(GGAA)	GAAC	0.02

Table S2
Effects of P5c-P2 interactions on observed rate constants of the bimolecular ribozymes.

P2 in ΔP5 ribozyme	P5c in P5abc RNA	k_{obs} (min^{-1})
WT	WT	0.32
M5	M5	0.31
M4	M4	0.16
M3	M3	0.03
M4	M5	0.069
M5	M4	0.076
